# Epigenetics of conotruncal congenital heart disease: Protocol for a systematic review and meta-analysis

**DOI:** 10.1371/journal.pone.0302642

**Published:** 2024-04-30

**Authors:** Elhadi H. Aburawi, Linda Östlundh, Hanan E. Aburawi, Rami H. Al Rifai, Akshaya Bhagavathula, Abdelouahab Bellou

**Affiliations:** 1 Department of Pediatrics, UAE University, Al Ain, United Arab Emirates; 2 Örebro University Library, Örebro University, Örebro, Sweden; 3 Department of Biology, College of Sciences, UAE University, Al Ain, United Arab Emirates; 4 Institute of Public Health, College of Medicine & Health Sciences, UAE University, Al Ain, United Arab Emirates; 5 Department of Public Health, North Dakota State University, Fargo, ND, United States of America; 6 Department of Emergency Medicine, Institute of Sciences in Emergency Medicine, Guangdong Provincial People ‘s Hospital, Guangdong Academy of Medical Sciences, Guangzhou, China; 7 Department of Emergency Medicine, Wayne State University School of Medicine, Detroit, MI, United States of America; 8 Global Network on Emergency Medicine, Brookline, MA, United States of America; Human Genetics and Genome Research Institute, National Research Centre, EGYPT

## Abstract

**Background:**

Conotruncal congenital heart defects (CTD) are a subset of congenital heart diseases (CHD) that involve structural anomalies of the right, left, or both cardiac outflow tracts. CHD is caused by multifactorial inheritance and changes in the genes or chromosomes. Recently, CHD was found to be due to epigenetic alterations, which are a combination of genetic and other environmental factors. Epigenetics is the study of how a gene’s function changes as a result of environmental and behavioral influences. These causative factors can indirectly cause CHD by altering the DNA through epigenetic modifications. This is a protocol for a systematic review and meta-analysis that aims to explore whether the strength of association between various epigenetic changes and CTD types varies by race. Furthermore, to determine and compare the changes in gene expression of each mutation.

**Methods:**

Our protocol follows the Preferred Reporting Items for Systematic Reviews and Meta-Analyses Protocol (PRISMA-P) guidelines. A comprehensive pre-search has been developed in PubMed and PubMed’s Medical Subject Headings (MeSH). The final search will be performed in June 2023 in PubMed, Embase, Scopus, Web of Science, Cochrane Library, CIANHL, and PsycInfo, without restrictions on publication years. The Covidence systematic review software will be used for blinded screening and selection. Conflicts will be resolved by a third, independent reviewer. The risk of bias in selected studies will be assessed using the National Heart, Lung, and Blood Institute (NHLBI) Quality Assessment Tool for Observational Cohort and Cross-Sectional Studies. The data to be extracted will cover basic information on the included studies, study sample size, number of patients with various types of epigenetic changes, number of patients with various CTD types, measures of association and their 95% confidence interval between each epigenetic change and each CTD. The protocol has been registered with the International Prospero Register of Systematic Review (PROSPERO) [CRD42023377597].

**Discussion:**

To the best of our knowledge, this protocol outlines the first systematic review and meta-analysis of the epigenetics of CTD. There is a growing body of evidence on epigenetics and its indirect involvement in disease by altering the DNA through epigenetic modifications in the genes associated with the causative factors for CHD. We will conduct a comprehensive and systematic search for literature in the above-mentioned seven core biomedical databases. It is very important to identify population-specific risk factors for CHD, which will have significant creative, custom-made, and effective prevention programs for the future generation.

## Introduction

Congenital heart disease (CHD) is the most common birth defect worldwide and the number one killer of live-born infants [1]. Conotruncal congenital heart defects (CTD) are a group of cardiac malformations that involve structural abnormalities of the right, left, or both cardiac outflow tracts (OFT). These are double outlet right ventricle (DORV), tetralogy of Fallot (ToF), ventricular septal defect (VSD), atrioventricular septal defect (AVSD), coarctation of the aorta (CoA), interrupted aortic arch (IAA) and atrial septal defect (ASD). The OFT, including the conus or bulbus cordis and the truncus arteriosus (together called the conotruncal part), is a rapidly remodeling structure during embryogenesis at the arterial end of the heart, as it connects the embryonic ventricles to the aortic sac [2]. It is well understood that cardiac anomalies are caused by multifactorial inheritance, including chromosomal defects, and environmental factors such as parental stress and medications used by the mother during the first trimester [3, 4]. Recently, it has become well understood that CHD is caused by the dysregulation of genetic and epigenetic factors [5, 6]. As a result, the role of epigenetic mechanisms in the causes of CHD is becoming more widely accepted [7–10]. The epigenome is the collection of chemical marks on the DNA and its associated proteins that determine how much and when genes are expressed. Epigenetics is the study of the mechanisms that cause changes in how genes function as a result of the effects of the organism’s behaviors and the environment, which can affect genetic expression and cellular differentiation [11]. These epigenetic modifications are reversible and independent of DNA sequence; they can change how the body reads a DNA sequence, altering its expression. The most recent evidence suggests that the aberrant regulation of gene expression by erroneous epigenetic mechanisms is the main factor that attracts attention to its role in the development of CHD. DNA carries these reversible epigenetic changes, which usually occur through three established mechanisms [11]:

### DNA methylation and demethylation

These are the processes by which a chemical group, a methyl group, is added to (methylation) or removed from (demethylation) the fifth carbon of the nucleotide cytosine on specific places of the DNA sequence. Typically, this chemical group can physically interact with proteins that attach to DNA to read the gene and predominantly turn their expression off (methylation) or on (demethylation), although the exact effect definitely varies with context.

- **Histone modification**: DNA is packed around histone proteins to make up structures called chromatin. The modification of histones through the addition, or potential removal, of chemical groups such as methyl leads to tightly or loosely wrapped chromatin, allowing varying access of regulatory elements and proteins to the DNA. Subsequently, these chemical groups change whether a gene is wrapped or unwrapped, thereby initiating, increasing, decreasing, or halting its expression [12]. Aberrant expression and mutations of the histone modifiers during the development of the heart can influence the response of the heart to pathological stresses [3].
- **Non-coding RNA:** DNA is generally used as a blueprint for creating coding and non-coding RNAs (ncRNA), and while the latter is not translated into proteins, it can have other regulatory functions, such as helping to control gene expression. This regulation of gene expression can be achieved by using ncRNA, along with certain proteins, to breakdown coding RNAs so that they cannot be used to make proteins, leading to their diminished expression. NcRNAs may also employ proteins to change histones to turn genes on or off. In addition, mRNA, rRNA, and tRNA can also undergo modifications that affect gene expression.

### Objectives

1. To explore whether the strength of association between various epigenetic changes and CTD types is varied by race. 2. To determine the gene expression through upregulation and downregulation of each mutation in the three tools of investigation studied. 3. This systematic review, using meta-analysis, will also summarize the strength of association between various types of epigenetic changes; DNA methylation, histone modification, chromatin remodeling, and ncRNAs, with the reported CTD types.

## Methods

This protocol followed the Preferred Reporting Items for Systematic Reviews and Meta-Analyses Protocol guidelines (PRISMA-P) [13]. It is registered with the International Prospective Register of Systematic Reviews (PROSPERO) [CRD42023377597].

### Eligibility criteria

All peer-reviewed published observational, retrospective, and prospective cohort studies in humans in the English language will be included. All animal studies, all types of reviews, editorials, case reports, conference abstracts, opinions, letters to the editors, literature reviews, clinical trials, comments, studies in languages other than English, grey literature, opinions, pre-prints, and other interventional studies will be excluded.

### Study design

The final review will be informed by the Cochrane Handbook for Systematic Reviews of Interventions [14] and reported in accordance with the PRISMA statement, including the PRISMA-S extension for searching [13, 15].

The Covidence systematic review software is to be used for deduplication, blind screening, and selection by two independent subject specialists. Conflicts will be resolved by a third, independent reviewer [16]. The risk of bias in selected studies will be assessed using the NHLBI Quality Assessment Tool for Observational Cohort and Cross-Sectional Studies [17, 18].

### Population

We will include international studies of all patients with CTD who underwent epigenetic and genomic studies, and we will exclude any other studies of CHD.

### Exposure and outcome

Various epigenetic alteration types will be the subject of interest. Epigenetic alteration types will include DNA methylation, acetylation, histone modification, chromatin remodeling, and ncRNA. Those with no epigenetic alterations will be considered a collective control group for comparison.

We will find out how epigenetic changes affect the DNA that makes up the genes of CTD patients with new mutation(s). We want to learn about the relationship between epigenetic errors and the DNA sequence in those CTD patients from different racial origins. For example, whether a methyl group is added to or removed from specific places on a DNA sequence depends on the specific CTD, aberrant expression, and specific mutations of histone modifiers during the development of the heart that can influence the response of the heart to any pathological process. Furthermore, RNA can undergo modifications that affect gene expression through post-transcriptional regulation mechanisms in certain CHDs.

### Information sources

The literature will be identified by systematically searching the electronic biomedical databases PubMed (NML), Embase (Elsevier), Scopus (Elsevier), Web of Science (Clarivate), CIANHL (EBSCOhost), and PsycInfo (EBSCOhost). In addition, the references list of the finally selected articles will also be systematically screened manually for additional potential articles. The search will rule out any observational studies that are not peer-reviewed, unpublished, or conducted in organisms other than humans. Cabell’s Predatory Reports [19] will be consulted to verify the non-predatory status of selected papers published in open-access journals. The search will be performed in June 2023, with a full search update in the manuscript preparation phase.

### Search strategy

A preliminary strategy using PubMed was developed in December 2022- March 2023 by a medical librarian specializing in systematic review methodology and searching (LÕ). The search terms were systematically selected based on PubMed’s Medical Subject Headings (MeSH) and by analyzing key publications in the field. The final selection of search terms was reviewed and confirmed by the subject specialists (EHA & HEA) after a pilot screening of selected results from the preliminary search.

A combination of the search fields “Title”, “Abstract”, “Keywords” (i.e., Text Word and Topic) and the MeSH/Thesaurus (when available) will be used for all search terms to ensure that the best possible evidence is located and to include potential pre-indexed materials. The search will be performed without publication or year restriction and is limited to include English language studies only. The search string developed for PubMed will be repeated in all databases in May 2023. Finally, systematic, manual screening of the reference lists of the included studies will be conducted (HEA).

As already mentioned above, the search will be guided by the PRISMA-S extension, and a search log with all technical search details, search term inclusions, results, dates, and notes for all included sources will be appended to the review [13, 15]. Cabell’s Predatory Reports [19] will be consulted to verify the non-predatory status of selected papers published in open-access journals. A reproducible search string and results from the pre-search performed in PubMed on March 6, 2023, are available in online supplementary material 1.

### Data management

All records located in the literature search will be exported to the systematic review software Covidence [16] for automatic de-duplication and blinded screening, selection, and conflict resolution. The final records will be transferred to the Zotero reference management tool as support for manuscript preparation.

### Study selection process

The Covidence systematic review software will be used for deduplication and full blinding of the selection and conflict resolution processes. Two independent reviewers (EHA & HEA) will initially screen the titles and abstracts of all unique records located in the database search. A third reviewer (AB) will resolve any eventual conflicts identified by the software. Full text for the studies selected as potentially eligible in the title and abstract screening will be obtained and uploaded to Covidence for blind screening by two reviewers (EHA & HEA). A third reviewer (AB) will resolve the final conflicts [16]. The details for the screening and selection process will be presented in a PRISMA flow diagram [18, 20] in final review.

### Data collection process

Two independent reviewers (EHA & HEA) will use the Covidence software to extract data from the final selected studies. A third researcher (AB) will review the details and resolve eventual conflicts. A pilot including a minimum of eligible studies will be conducted to confirm the final design of the data extraction sheet ahead of the final extraction. Basic study characteristics such as author names, publication years, sources, and locations (country in which the research was conducted) will be extracted and recorded. The extracted data on current knowledge on epigenetic alterations associated with CTD will be recorded. The results of epigenetic investigations and their association with each of the above-mentioned CTD types will be recorded. The variables on epigenetic changes in each of the CTD across different racial groups will also be recorded. Any upregulations and downregulations of gene expression in any mutation in the above tools of studied investigations will be registered.

### Meta-analysis

Whenever applicable and enough data is available from primary studies, meta-analysis will be performed to summarize the overall strength of association between epigenetic changes and CTD types. The variation in this strength of the association will also be explored by race. Pre-calculated effect estimates (odds ratio, relative risk, and rate ratio) will be pooled together by the type of estimate according to the study design. Estimates reported in cross-sectional studies will be pooled independently of estimates reported in cohort studies.

### Risk of bias assessment

The risk of bias and quality of evidence in the eligible studies will be evaluated by two independent reviewers (EHA & HEA) using the NHLBI Quality Assessment Tool for Observational Cohort and Cross-Sectional Studies [21]. The template of the tool will be uploaded to Covidence systematic review software for blinded assessment. The quality assessment will include a critical analysis to identify potential publication, selection, outcome, or conclusion biases in the selected studies [19]. A third reviewer (AB) will independently resolve any eventual conflicts identified by the software.

To minimize any potential bias in identifying eligible studies as well as to avoid any human error in data extraction, seven databases (PubMed, Embase, Scopus, Web of Science, Cochrane Library, CIANHL, and PsycInfo) will be searched. Eligible studies will be extracted, and their quality will be assessed independently by two reviewers.

## Discussion

This protocol outlines the first systematic review and meta-analysis on the epigenetics of CTD, to the best of our knowledge. The quality of the evidence reported will be supported by comprehensive and systematic searches for literature in seven core biomedical databases and a transparently reported, blinded screening, conflict resolution, extraction, and quality assessment process. Although we do not intend to modify the protocol after it has been submitted, any eventual minor amendments made during the review process will be transparently recorded with dates and details in the online PROSPERO registration to avoid publication bias.

The main limitation of the study is that there could be multiple confounding factors, such as maternal obesity and diabetes mellitus, for which we could not predict or conclude their effects on the mechanism of CHD. Furthermore, genetic sequencing will probably elucidate the incomplete understanding of the etiology of CHD. In view of the changing epidemiology of CHD, plus the unknown survival rate and wide range of reported birth prevalence, the estimates of the number of CHD could be more complicated and inaccurate [22]. The dissemination plan is to publish our findings and compare them with other studies in this field in the hopes of finding new modalities for prevention and treatment. In terms of future directions and future studies, the main challenges for scientists are refining and assimilating, or conforming, vital research data on epigenetic amendments in heart development and CHD. This would be with the hope of identifying consistent and explicit epigenetic biomarkers and launching epigenetic-based prevention and treatment programs for CHD.

### Review status

A preliminary search was performed in PubMed with a start date of December 2022, and the result was updated in March 2023 (see online S1 File). The review is set to start in June 2023.

## Supporting information

S1 Checklist PRISMA-P (Preferred Reporting Items for Systematic review and Meta-Analysis Protocols) 2015 checklist: Recommended items to address in a systematic review protocol*. (DOC)

S1 File Search results pre search in PubMed on Feb. 21, 2023. (DOCX)
