# Supplementary material for: Epigenetics of conotruncal congenital heart disease: Protocol for a systematic review and meta-analysis
Source: PLoS One. 2024 Apr 30;19(4):e0302642. doi: 10.1371/journal.pone.0302642 (PMC11060528; doi:10.1371/journal.pone.0302642)
Supplement: S1 File — (DOCX) [file pone.0302642.s002.docx]

**Supplementary material 1**

**Preliminary literature search in PubMed 2023-03-27**

| **Source and search date** | **Search string** | **Results and notes** |
| --- | --- | --- |
| **PubMed**  (NML)  **Coverage:** from database inception to March 27, 2023 | (("Epigenomics"[MeSH] OR "Epigenesis, Genetic"[Mesh] OR "epigenetic*"[Text Word] OR "epigenesis"[Text Word] OR "epigenomic*"[Text Word]) AND ("Conotruncal*"[Text Word] OR “congenital heart malformation*”[Text Word] OR “congenital heart defect*”[Text Word] OR "heart defects, congenital"[MeSH:noexp] OR “right ventricle outflow” [Text Word] OR “left ventricle outflow”[Text Word] OR “atrial septal defect” [Text Word] OR "22q11 Deletion Syndrome*"[Text Word] OR "Deletion Syndrome, 22q11"[Text Word] OR "Deletion Syndromes, 22q11"[Text Word] OR "Syndrome, 22q11 Deletion"[Text Word] OR "Syndromes, 22q11 Deletion"[Text Word] OR “chromosome deletion 22q11” [Text Word] OR "Kousseff syndrome"[Text Word] OR "Genito palato cardiac syndrome"[Text Word] OR "Genitopalatocardiac syndrome"[Text Word] OR "Gardner-Silengo-Wachtel syndrome"[Text Word] OR "Genito-palato-cardiac syndrome"[Text Word] OR "DiGeorge Syndrome"[Text Word] OR "DiGeorge Syndrome"[MeSH Terms] OR "DiGeorge Sequence" [Text Word] OR "Pharyngeal Pouch Syndrome"[Text Word] OR "Autosomal Dominant Opitz G-Bbb Syndrome"[Text Word] OR "Autosomal Dominant Opitz G Bbb Syndrome"[Text Word] OR "Pharyngeal Pouch Syndrome"[Text Word] OR "Thymic Aplasia Syndrome"[Text Word] OR "Catch22"[Text Word] OR "DiGeorge Anomaly"[Text Word] OR "Hypoplasia of Thymus"[Text Word] OR "Velocardiofacial Syndrome"[Text Word] OR "Syndrome, Velocardiofacial"[Text Word] OR "Sedlackova Syndrome"[Text Word] OR "Syndrome, Sedlackova"[Text Word] OR "Shprintzen Syndrome"[Text Word] OR "22q11.2DS"[Text Word] OR "VCF Syndrome"[Text Word] OR "Velo-Cardio-Facial Syndrome"[Text Word] OR "Velo Cardio Facial Syndrome"[Text Word] OR "Deletion 22q11.2 Syndrome"[Text Word] OR "22q11.2 Deletion Syndrome"[Text Word] OR "Deletion Syndrome, 22q11.2"[Text Word] OR "Shprintzen VCF Syndrome"[Text Word] OR "22q11 Deletion Syndrome"[MeSH Terms] OR "Conotruncal cardiac defects"[Supplementary Concept] OR "Sacral meningocele conotruncal heart defects"[Supplementary Concept] OR "tetralogy of fallot*"[Text Word] OR "Tetralogy of Fallot"[Mesh] OR "Tetralogy, Fallot*"[Text Word] OR "Fallot's Tetralogy"[Text Word] OR "Fallot Tetralogy"[Text Word] OR "Fallots Tetralogy"[Text Word] OR "Truncus Arteriosus"[Text Word] OR "Truncus Arteriosus"[Mesh] OR "Truncus Arteriosus, Persistent"[Mesh] OR "Interrupted Aortic Arch*"[Text Word] OR "Aortic Coarctation"[Mesh] OR "Aortic Coarctation*" OR "Coarctation of the Aorta"[Text Word] OR "Coarctation of Aorta"[Text Word] OR "Aorta Dominant Coarctation*"[Text Word] OR “arterial trunk” [Text Word] OR "Transposition of the Great Arteries"[Text Word] OR "Congenitally Corrected Transposition of the Great Arteries"[Mesh] OR "Congenitally Corrected Transposition of the Great Vessel*" [Text Word] OR "Transposition of Great Vessel*"[Text Word] OR "Congenitally Corrected Transposition*"[Text Word] OR "Transposition of Great Vessels"[Mesh] OR "Great Vessels Transposition*"[Text Word] OR "Dextro-Looped Transposition of the Great Arteries" [Text Word] OR "Transposition of Great Arteries"[Text Word] OR "Great Arteries Transposition*"[Text Word] OR "Double Outlet Right Ventricle*"[Text Word] OR "Double Outlet Right Ventricle"[Mesh] OR "Double-Outlet Right Ventricle*"[Text Word] OR "Right Ventricle, Double-Outlet"[Text Word] OR "Ventricle, Double-Outlet Right" [Text Word] OR "Taussig-Bing Anomaly" [Text Word] OR "Taussig Bing Anomaly" [Text Word] OR "Hypoplastic Left Heart Syndrome*"[Text Word] OR "Hypoplastic Left Heart Syndrome"[Mesh] OR "Left Heart Syndrome, Hypoplastic" OR "Bicuspid Aortic Valve*"[Text Word] OR "Bicuspid Aortic Valve Disease"[Mesh] OR "Aortic Valve, Bicuspid"[Text Word] OR “Aortic valve stenosis”[Text Word])) | **Results: 411**  **Notes:**  All keywords are searched in the fields: “title” and “abstract” and in MeSH when available.  The search is limited to English studies only. |
